# Shock Wave Pressure Measurement and Calibration Method Based on Bar Pressure Sensor

**DOI:** 10.3390/s25154743

**Published:** 2025-08-01

**Authors:** Yong-Xiang Shi, Ying-Cheng Peng, Yuan-Ding Xing, Xue-Jie Jiao, Xiao-Fei Huang, Ze-Qun Ba

**Affiliations:** Northwest Institute of Nuclear Technology, Xi’an 710024, China; 17699686137@163.com (Y.-X.S.); j01307@sohu.com (Y.-C.P.); 18196448306@163.com (Y.-D.X.); fei.hx@163.com (X.-F.H.); b15098722652@163.com (Z.-Q.B.)

**Keywords:** bar pressure sensor, shock wave, strain measurement

## Abstract

In order to correctly measure the shock wave pressure generated by a near-field explosion, and while considering the limitations of the measurement and calibration method of the current bar pressure sensor, an improved shock wave pressure measurement method was designed based on a bar pressure sensor combined with photon Doppler velocimetry (PDV) and strain measurement. By measuring the strain on the pressure bar and the particle velocity on the rear-end face, the shock wave pressure applied on the front-end face of the pressure bar was calculated based on one-dimensional stress wave theory. On the other hand, a calibration method was designed to validate the reliability of the test system. Based on the split-Hopkinson pressure bar (SHPB) loading experiment, the transmission characteristics of stress wave in the bar and the accuracy of the system test results were verified. The results indicated that the stress wave measurement results were consistent with the one-dimensional elementary theoretical calculation results of stress wave propagation in different wave-impedance materials, and the peak deviation measured by PDV and strain measurement method was less than 1.5%, which proved the accuracy of the test method and the feasibility of the calibration method.

## 1. Introduction

Explosives produce chemical reactions in a very short time and in a very small range, instantly generating a large amount of high-temperature and high-pressure gas. When the high-temperature and high-pressure gas reaches the surrounding medium, shock waves will be generated. The amplitude of the shock wave pressure is one of the main indicators for measuring and evaluating the degree of damage to the human body and the surrounding environment. Therefore, the measurement of shock wave pressure in a near-field explosion is of great significance to damage efficiency research, weapon equipment development, engineering safety assessment, and so on.

The shock wave pressure generated in a near-field explosion has a large amplitude, is fast rising, has a short pulse, and creates serious electromagnetic interference. It is also highly demanding for sensors to completely measure and record the shock wave pressure waveform. At present, the most commonly used sensors which measure the impact pressure include piezoresistive sensors, piezoelectric sensors, and polyvinylidene fluoride (PVDF) pressure sensors. The piezoresistive shock wave pressure sensor possesses good low-frequency characteristics, but it is sensitive to light radiation and thermal radiation, and it is not suitable for damage conditions with a strong fire light, significant temperature change, or a strong ionization field. The piezoelectric shock wave pressure sensor has good dynamic characteristics, but its low-frequency characteristics are not as good as that of the piezoresistive sensor [1,2], and it is susceptible to interference on occasion from high electromagnetic radiation or a long test distance. The PVDF pressure sensor has the advantages of small size, thin thickness, strong structure, good impact resistance, high sensitivity, fast dynamic response, and good matching performance with the medium, but its pyroelectric coefficient is large, the measurement accuracy is susceptible to temperature changes [3], and there are no mature commercial products currently.

The Hopkinson bar pressure sensor is a kind of sensor which uses the pressure bar as an elastic sensitive element to realize the conversion of pressure to stress, strain, and other mechanical quantities. It was first proposed by Hopkinson [4] in 1914. Early research was mainly focused on how to obtain the pressure–time history curve [5,6], improve the frequency response, and extend the effective measurement time [7,8]. With the development of the equipment, research was gradually applied to the field of explosion shock wave testing. In 1996, Dick et al. [9] successfully measured the shock wave pressure of 2.4 GPa peak value in a near-field explosion by using a pressure bar.

In China, Hu Yongle, Shi Peijie, and Zhang Dezhi from the Northwest Institute of Nuclear Technology [10,11,12] designed and analyzed the pressure bar pressure sensor system. Yi et al. and Yang [13,14] carried out influence factor analysis and test method research on the whole pressure bar test system, optimized the sensor design and test analysis system, and proved through testing that the bar pressure sensor played an irreplaceable role in the research of explosion shock wave testing due to its advantages such as its large range, high-frequency response, and high durability of sensitive elements.

For shock wave pressure measurement on the basis of a bar pressure sensor, the strain measurement is the most commonly adopted method at present, which is easily affected by the parasitic effect and electromagnetic interference, and there are few mature calibration methods for the test system. In the field of the pressure measurement system (PMS), the shock tube and split-Hopkinson pressure bar (SHPB) [15] have both played an important role in calibration. The shock tube generates a shock wave when the diaphragm ruptures or a valve opens so that if high-pressure gas expands into a low-pressure gas section [16,17,18], a high-pressure jump and a high-temperature jump is produced in a short time, where the jump can be an almost ideal step change in the range of ns [19]. Meanwhile, the shock tube has weaknesses such as vulnerability to environmental interference, possessing a costly diaphragm, exhibiting uncertainty in calibration, and so on [20,21]. The SHPB is widely used in the field of experimental mechanics, and it has the advantages of strong environmental adaptability, low cost, and good repeatability of test results [15,22]. Due to the demand of shock wave pressure measurement for a near-field explosion and the limitations of the measurement and calibration method of a bar pressure sensor, this paper designs a shock wave pressure measurement method based on the bar pressure sensor combined with PDV [23] measurement and strain measurement. The pressure bar is used as a mechanical sensitive element to respond to the pressure of the reflected shock wave, which is converted into strain and particle velocity measurements on the bar. The calibration method is designed and both the transfer characteristics of the stress wave in the bar and the accuracy of the test results are verified based on the SHPB loading experiment.

## 2. Materials and Methods

### 2.1. Bar Pressure Sensor Design

The material and structure design of bar pressure sensors are important factors that affect measurement results. The mechanical properties of the pressure bar determine the sensor sensitivity, measuring range, frequency response characteristics, and other parameters.

#### 2.1.1. Selection of the Pressure Bar Materials

Studies have shown that pressure bars made of materials with a small Poisson’s ratio and high dynamic yield strength will have better performance [14]. A carbon tool steel with a low Poisson’s ratio and high strength was selected as the material of the pressure bar to reduce the transverse inertia effect and ensure that the pressure bar worked in the elastic stage at full scale.

#### 2.1.2. Design of the Pressure Bar Diameter

To satisfy the one-dimensional stress wave assumption, the measurable wave length in the bar with a radius of r should satisfy r/λ_m_ ≤ 0.1; the frequency of the explosion shock wave to be measured was not higher than 100 kHz, and the elastic wave velocity $C_{0}$ took the value of 5170 m/s. It could be obtained that λ_m_ = 0.0517 m and r ≤ 5.17 mm. In accordance with the elementary theory, smaller diameter bars could effectively reduce the rise time and had better high-frequency characteristics; the relationship between rise time and bar diameter (D), the distance from the measuring point to pressure end (z), and the Poisson‘s ratio (σ) is expressed as follows [24]:

(1) $$\tau =\frac{D}{C_{0}}*\sqrt[{3}]{\sigma^{2}z/D}$$

In order to reduce the rise time, improve the high-frequency characteristics, and make it easy to process, the diameter of the pressure bar was designed to be 4 mm and 6 mm, and the rise time was calculated to be 2.1 μs and 3.1 μs, respectively.

#### 2.1.3. Design of the Pressure Bar Length

The length of the pressure bar affects the effective measurement time. In general, the length of the pressure bar is determined after estimating the positive pressure time of the pressure pulse according to the test parameters. The length of the pressure bar should be adapted to the time scale of the measured pressure signal to ensure that all positive pressure peak intervals could be gained, and the signal duration was generally within 100 microseconds. When PDV is used to measure the velocity of free-end face particles, the effective measurement time is the time interval from the take-off point of velocity to the beginning of elastic wave superposition; that is, the time as the elastic wave propagates back and forth in the rod once. The effective measurement time is as follows:

(2) $$t=\frac{2L_{0}}{C_{0}}$$

In the formula, t is the propagation time, $C_{0}$ is the elastic wave velocity, and $L_{0}$ is the bar length. To meet the requirements, this design took two bars whose length is 1000 mm and 1500 mm, respectively, and the effective measurement time that could be satisfied is 387 μs and 580 μs, correspondingly. The performance index of pressure bar is shown in Table 1.

### 2.2. Shock Wave Pressure Measurement System Based on the Bar Pressure Sensor

**Figure 1:**
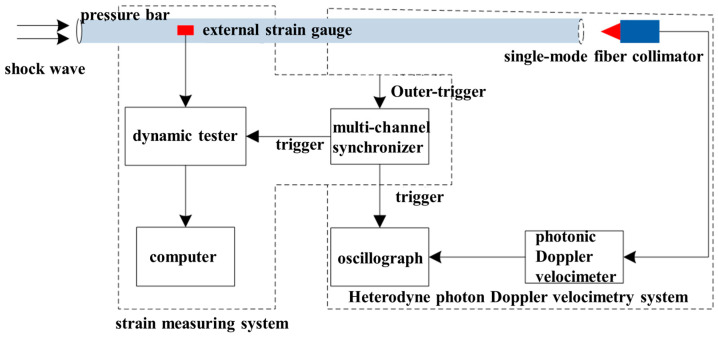
Shock wave pressure measurement system.

By combining contact and local point electrical measurement technology with non-contact, high-precision, full-field, and high spatial and temporal resolution optical measurement technology, a shock wave pressure measurement system based on the bar pressure sensor was designed by using the strain measurement method and heterodyne PDV measurement method, as shown in Figure 1.

The strain measurement system was mainly composed of an external strain gauge (sensor), dynamic tester (measurement and analysis), multi-channel synchronizer (trigger), and a computer. In order to suppress the influence of parasitic effects such as cable length and temperature on the strain measurement, the full-bridge method was used to measure the stress and deformation of the pressure bar. So as to eliminate the interference of non-axial strain, two signals were measured on the same pressure bar; that is, two working strain gauges were symmetrically attached to both sides of the pressure bar and placed on the same cross-section of the pressure bar to ensure the synchronization of the measured signals.

The heterodyne PDV system was mainly composed of a single-mode fiber collimator, a photonic Doppler velocimeter, a multi-channel synchronizer (trigger), and an oscilloscope. The free surface particle velocity of the rear-end face of the measuring bar was measured by the PDV.

**Figure 2:**
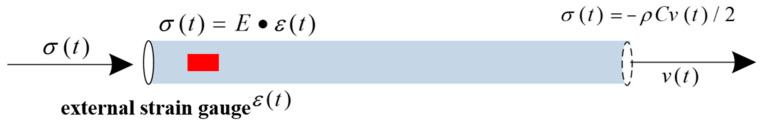
Diagram of the pressure measurement principle and the transformation relationship between measurement parameters.

When measuring the shock wave impact, the two systems triggered measurement at the same time to refer to each other. The pressure measurement principle of the bar pressure sensor is shown in Figure 2. The transformation relationship [25] between the velocity of the free-end face of the pressure bar and stress, strain is as follows:

(3) $$\sigma (t)=-\rho C_{0}v(t)/2$$

(4) $$v=-2C_{0}\varepsilon$$

### 2.3. Calibration Test System

**Figure 3:**
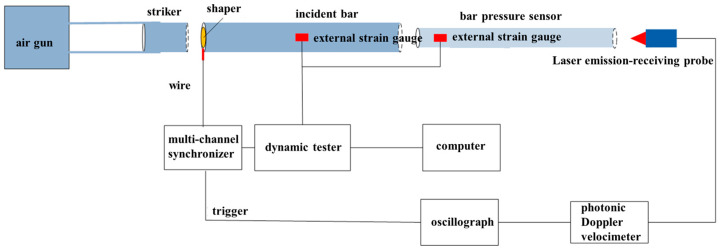
Calibration and test system for the bar pressure sensors based on the split-Hopkinson pressure bar.

With the intention of verifying the accuracy of the shock wave pressure measurement system, a bar pressure sensor calibration test system based on the split-Hopkinson pressure bar was designed, as shown in Figure 3. A cylindrical bullet was shot from a high-pressure gas chamber to impact the incident bar at a certain speed, generating an incident wave signal. As the signal propagated to the bar pressure sensor, reflection and transmission would be generated at the contact-end face (i.e., the variable cross-section bar). At this time, there were incident waves and reflected waves in the incident bar, and there were transmitted waves in the bar pressure sensor. The strain of the incident bar and bar pressure sensor as well as particle velocity on the end face of the pressure bar were measured, and the incident wave, reflected wave, and transmitted wave were compared to verify the transmission characteristics of a stress wave in different wave-impedance materials. The self-consistency of each of the two test methods was acquired by comparing the strain and particle velocity measurement results in the pressure bar.

## 3. Results and Discussion

### 3.1. Experimental Overview

The diameter of the striker and incident bar of the split-Hopkinson pressure bar device was 30 mm, and the length was 200 mm and 2000 mm, respectively. They were all made of 34GrMnSiA high-strength steel, and the wave velocity was about 5186 m/s.

The brass sheet was selected as the shaper to filter the high-frequency components caused by direct collision, reducing the dispersion distortion of the wave in long-distance propagation process, and to make the rising edge of the incident wave flatter. By adjusting the diameter of the shaper or the loading speed, it could be ensured that the reflected wave was basically a platform wave in order to achieve constant strain rate loading.

At which time the striker hit the incident bar, the two test systems were triggered to record simultaneously. The result measured by the strain measurement system was the signal of the strain changing with time. The result measured by the PDV system was the signal of the voltage changing with the time of the interference mixing. The time–frequency signal curve could be derived by Fourier synchronous squeezing transformation, and then the curve of particle velocity changing with time could be obtained by conversion.

### 3.2. Experimental Results and Analysis

**Figure 4:**
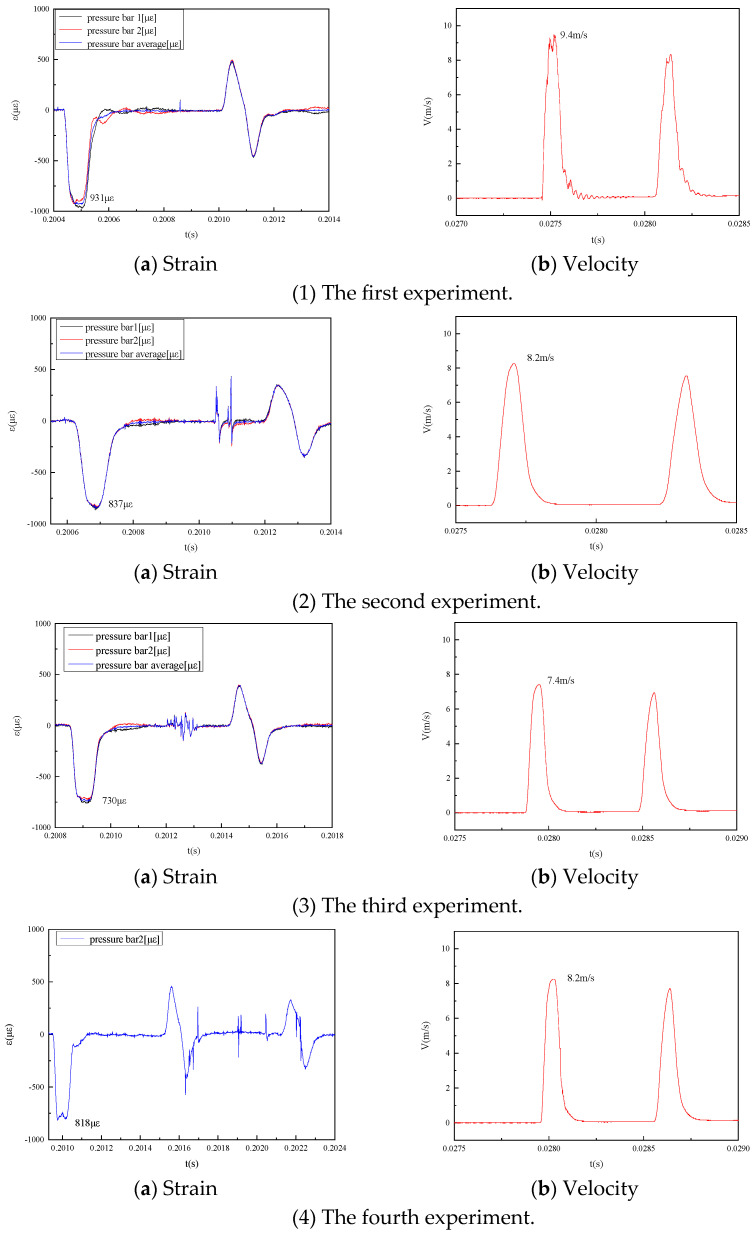
Analysis results of the four strain and velocity measurements.

A total of four verification experiments were carried out, and the results of the strain and velocity tests are shown in Figure 4.

In light of the difference between take-off time of the first peak and the eleventh peak of the measured results, the propagation velocity of each wave in the different materials was determined by Formula (5):

(5) $$C=\frac{10\cdot 2\cdot L}{\Delta t}$$

By calculation, the average wave velocity in the incident bar and transmission bar was 5186 m/s and 4976 m/s, respectively, and then the relative deviation of velocity results of the different experiments was derived by Formulas (3) and (4), as shown in Table 2. Compared with the traditional measurement method (i.e., strain measurement), the peak deviation was less than 1.5%, which proved the feasibility of the two measurement methods.

To validate the transmission characteristics of the stress wave in different wave-impedance materials, the measurement results of the incident wave, reflected wave, and transmitted wave were compared with the theoretical calculation results. As soon as the strong discontinuity propagates from the medium in which the impedance is $\rho_{i}C_{i}$ to the medium in which the impedance is $\rho_{t}C_{t}$, the left-propagating reflected wave and the right-propagating transmitted wave would be generated at the interface of the two media. According to the continuity condition at the interface and Newton’s third law, it could be attained that the post-wave particle velocity, the total force of the reflected wave, and the transmitted wave at the consolidation interface should be equal. That is:

(6) $$\Delta v_{I}+\Delta v_{R}=\Delta v_{T}$$

(7) $$A_{i}\left(\Delta \sigma_{I}+\Delta \sigma_{R}\right)=\Delta \sigma_{T}A_{t}$$

Based on the equation for the conservation of wave front momentum, the Formula (6) above can be simplified as follows:

(8) $$\frac{\Delta \sigma_{I}}{{\left(\rho_{0}c_{0}\right)}_{1}}-\frac{\Delta \sigma_{R}}{{\left(\rho_{0}c_{0}\right)}_{1}}=\frac{\Delta \sigma_{T}}{{\left(\rho_{0}c_{0}\right)}_{2}}$$

and simultaneously (7):

(9) $$\left\{\begin{array}{c} \Delta \sigma_{R}=F\cdot \Delta \sigma_{I}\\ \Delta v_{R}=-F\cdot \Delta v_{I} \end{array}\right.$$

(10) $$\left\{\begin{array}{c} \Delta \sigma_{T}=T\cdot \Delta \sigma_{I}\frac{A_{i}}{A_{y}}\\ \Delta v_{T}=nT\cdot \Delta v_{I} \end{array}\right.;$$

in which,

(11) $$\left\{\begin{array}{c} n=\frac{{\left(\rho_{0}c_{0}A\right)}_{1}}{{\left(\rho_{0}c_{0}A\right)}_{2}}\\ F=\frac{1-n}{1+n}\\ T=\frac{2}{1+n} \end{array}\right.$$

**Figure 5:**
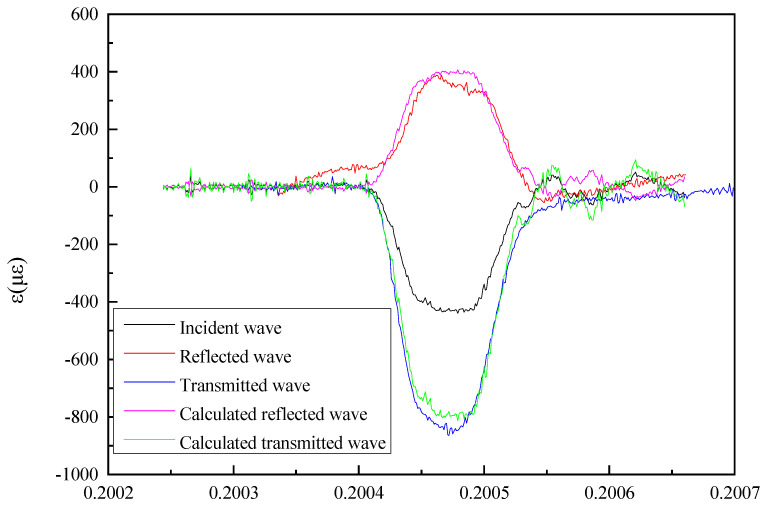
Comparison between the measured results and theoretical calculation results of the reflected and transmitted wave.

By substituting the material parameters of the incident bar and the transmission bar, the reflected wave and the transmitted wave were calculated as Figure 5 shows.

Figure 5 shows that the measured results were very close to the theoretical results, indicating that the calibration test system conformed to the one-dimensional elementary theory, which verifies the accuracy of the system and provides theoretical support for the system’s design. It further provides a reference for many scholars to study how to calibrate designed bar pressure sensor.

## 4. Conclusions

The results of the loading verification experiments and theoretical calculation based on SHPB demonstrated the following:

(1) In the designed shock wave pressure measurement system where a bar pressure sensor was combined with PDV and strain measurement, the velocity deviation obtained by PDV and strain measurement was less than 1.5%, which proved the self-consistency of the two test methods.

(2) The experimental results of the stress wave measurement were consistent with the one-dimensional elementary theoretical calculation results of the stress wave propagation in different wave-impedance materials, which proves the accuracy of the test method and the feasibility of the calibration method.

The performance index of the bar pressure sensor combined with the PDV sensor could be adjusted according to demands, while the equipment itself has low costs, which is suitable for the measurement of shock wave pressure generated by a near-field explosion.

## Figures and Tables

**Table 1:** Performance index of the pressure bar.

Material	Diameter (mm)	Length (mm)	Elastic Modulus (GPa)	Poisson’s Ratio σ	Density (g/cm^3^)	Wave Velocity (m/s)	Rise Time (μs)	Measurement Time (μs)	Measuring Range (MPa)
SKH51	4	1000	207.35	0.274	7.8	5170	2.1	387	785
	6	1500					3.1	580	

**Table 2:** Comparison results of different experiments.

Experimental Number	Mean Strain (με)	Reduced Velocity (m/s)	Calculated Pressure (MPa)	Measured Velocity (m/s)	Relative Deviation (%)
1	931	9.3	179.8	9.4	1.07
2	837	8.3	161.7	8.2	1.20
3	730	7.3	141.0	7.4	1.37
4	818	8.1	158.0	8.2	1.23

## Data Availability

Data available upon request.
